# Addressing Structural and Systemic Racism in Social and Health Care Systems to Advance Health Equity

**DOI:** 10.1007/s11606-025-09951-3

**Published:** 2025-12-02

**Authors:** Marshall H. Chin, Amy Gyau-Moyer, Allison Kelliher, Antonia M. Villarruel

**Affiliations:** 1 Department of Medicine, University of Chicago, Chicago, IL USA; 2 National Academy of Medicine, Washington, D.C USA; 3 University of North Dakota School of Medicine and Health Sciences, Grand Forks, ND USA; 4 Penn Nursing, University of Pennsylvania, Philadelphia, PA USA

## Abstract

**Background:**

American Public Health Association, American Medical Association, and American Nurses Association have declared racism a public health crisis because systemic oppression harms the care of individuals and the health of populations.

**Objective:**

To help increase recognition, knowledge, and action to address structural racism contributing to health inequities.

**Design/Approach:**

American Public Health Association Press has published a book, *Systems That Impact Population Health: Past and Present,* that was written with support and coordination from the National Academy of Medicine Culture of Health Program funded by the Robert Wood Johnson Foundation. This article provides an overview of the book and its conceptual model and actionable solutions.

**Key Results:**

Structural and systemic racism decreases access to opportunity structures for economic vitality, education, housing, justice, and health care, and directly exposes individuals to health risks including chronic stress, discrimination, and stigma. Racism segregates communities, leading to cumulative disadvantage and worse health. Actionable solutions can address structural racism to advance health equity in health care systems and social systems such as economic infrastructure, labor and employment, education, justice and civil rights law, media, immigration and foreign policy, and data and information.

**Conclusions:**

Improving social and health care systems helps poor, marginalized white populations, people of color, and the nation overall.

## STRUCTURAL RACISM IN SOCIAL SYSTEMS SETS THE STAGE FOR HEALTH INEQUITIES, YET IS UNDERADDRESSED

While unequal treatment in the health care system is one cause of health inequities across racial and ethnic populations^1^, structural racism embedded in social systems sets the stage. Structural racism’s effects are sometimes obvious, such as physical violence by police against American Indian and Alaska Native (AIAN) peoples and Black and Latino persons, and anti-Asian violence during the COVID-19 epidemic in the context of stigmatizing language^2^. Other mechanisms of action are subtler, such as the overrepresentation of people of color in essential worker jobs at higher risk for COVID-19 exposure, inequitable distribution of COVID-19 vaccines, and barriers that prevented many persons of racial/ethnic minority groups from accessing well-resourced health care delivery organizations most capable of caring for severely ill patients in respiratory failure^3^. Yet, addressing structural racism has not been a major focus of efforts to improve population health and advance health equity, that is, providing everyone a fair and just opportunity to be healthy^2^. Moreover, interventions that address structural racism to advance health equity improve health care and social systems for all people.^4^

The *Ending Unequal Treatment* report defines structural racism as: “The totality of ways in which a society fosters racial and ethnic inequity and subjugation through mutually reinforcing systems, including housing, education, employment, earnings, benefits, credit, media, health care, and the criminal legal system. These structural factors organize the distribution of power and resources (i.e., the social determinants of health) differentially among racial, ethnic, and socioeconomic groups, perpetuating racial and ethnic health inequities. The key difference between institutional and structural racism is that structural racism happens across institutions, and institutional racism happens within institutions.”^1^

Addressing structural racism is part of all health care and public health professionals’ responsibilities because systemic oppression harms the care of individuals and the health of populations. Structural racism affects health and the issues people of color disproportionately face—including discrimination. Structural racism influences the policies that finance and pay for health care and the ways that clinics and hospitals operate, including who can be seen and how they are treated. In fact, the American Public Health Association, American Medical Association^5^, American Nurses Association, and Association of American Medical Colleges have declared racism a public health crisis and committed to action, including self-inspection, education, partnerships, research, and policy development to address racism. Understanding and addressing structural racism is very much the responsibility of health care and public health professionals.

Yet, the health care industry has been slow to discuss and tackle structural racism. Health disparities training for health care professionals has historically focused on cultural competency and awareness of implicit bias rather than systems change. Until recently, few quality improvement activities have been conducted with an equity lens, and health equity initiatives such as screening and referrals for health-related social needs frequently do not explicitly recognize or address structural racism^2,6^.

Communities face health inequities due to structural barriers and misaligned health system goals, worsened by structural racism^2^. A matter of life or death, these systemic inequities limit access to resources and care, leading to poorer health outcomes for marginalized groups. The goal of health systems should be to maximize the health and well-being of all persons and communities^7^. However, financial incentives in the US system encourage health care delivery organizations to care for high-reimbursing patients with private insurance rather than patients with Medicaid insurance or no insurance. Health care delivery organizations typically maximize revenue by performing expensive procedures, surgeries, and diagnostic tests on privately insured patients rather than addressing social drivers of health, implementing preventative measures, or promoting health equity^7^. Person-centered care that meets the medical and social needs of all patients should be the driving force in creating more equitable health^8^, and not just a slogan or superficial, performative box to check to fulfill regulatory mandates. Clinicians, staff, administrators, and policymakers must break down structural barriers that impede community well-being. These solutions lead to health care and social systems that benefit all, including White people and those who are economically disadvantaged.

However, federal and state actions in 2025 are erasing the history of racism and eliminating policies that have reduced the effects of racism on health inequities^9^. More than ever, the nation needs to understand structural racism’s effects on the health of the public and promising solutions.

## AMERICAN PUBLIC HEALTH ASSOCIATION PRESS BOOK* SYSTEMS THAT IMPACT POPULATION HEALTH: PAST AND PRESENT*

### Process

To help fill the void in recognition, knowledge, and action to address structural racism contributing to health inequities, the American Public Health Association Press has published a book, *Systems That Impact Population Health: Past and Present*^2^, that was written with the support and coordination from the National Academy of Medicine (NAM) Culture of Health Program funded by the Robert Wood Johnson Foundation (RWJF). In 2022, NAM formed seven interdisciplinary author groups corresponding to the 2024 Office of Management and Budget race/ethnicity categories—American Indian or Alaska Native (AIAN), Asian, Black or African American, Hispanic or Latino, Middle Eastern or North African (MENA), Native Hawaiian or Pacific Islander, White. In each chapter, subject matter experts, most of whom had lived experiences of their particular group, elucidated how structural racism in three to four social and health systems of their choice led to health inequities for their population group. They reviewed literature, incorporated community input, and outlined historical context. The first chapter introduced the series, and the last chapter described solutions across racial and ethnic groups, including disadvantaged White populations that address structural racism to advance health equity and uplift health for all. The process for developing this book intentionally deepened understanding and empathy among collaborators, resulting in more inclusive solutions that can improve health for all populations. This approach also amplified marginalized voices.

### Conceptual Model

**Figure 1:**
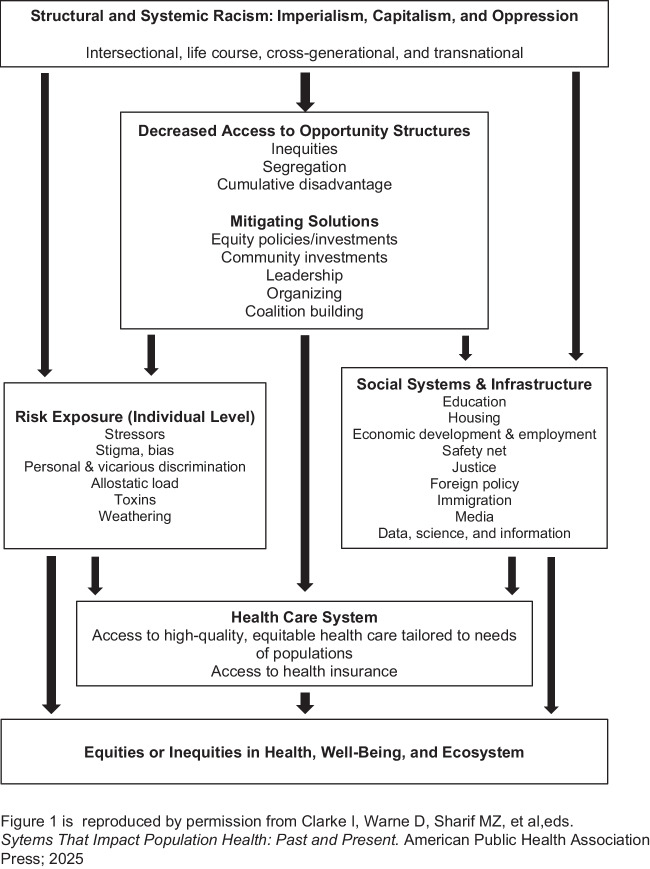
Conceptual model: structural and systemic racism’s impact on health equity/inequity.

Structural and systemic racism directly expose individuals to health risks, including chronic stress, discrimination, and stigma (Fig. 1). Opportunity structures provide access to beneficial resources, such as economic vitality, education, housing, justice, and health care. Structural and systemic racism decrease access to opportunity structures, segregating communities and leading to cumulative disadvantage and worse health^2^.

The book views structural and systemic racism within a larger intersectional lens that recognizes the synergistic effects of multiple systems of oppression, such as classism from unfettered capitalism, colonialism and its devastating effects on Indigenous peoples, and imperialism and its transnational effects^2^. History matters. Laws have been enacted over centuries to deliberately exclude groups from rights such as housing and voting. Devastating effects, such as the loss of generational wealth and limited access to education, remain for current populations.

The book also incorporates a lifecourse perspective and recognizes how intergenerational trauma from racism (e.g., slavery), colonialism (e.g., massacres of Indigenous peoples), and other oppressive systems sustain harm^2^. On average, Black children are exposed to adverse childhood experiences (ACES) much earlier than White children, with immediate and downstream damage to their health and opportunities^2^. AIAN children, unfortunately, experience higher numbers of ACES than persons from other groups, contributing to a milieu of health challenges^10^.

## STRUCTURAL RACISM EMBEDS INTO MULTIPLE SOCIAL AND HEALTH SYSTEMS

Structural racism is embedded into society’s fabric, and its effects cascade. Structural racism becomes insidiously invisible to many of those who blame persons of racial/ethnic minority groups for misfortunes. For example, structural racism caused discrimination and redlining in the housing and mortgage markets^2^, preventing many Blacks from owning homes and building wealth through housing equity. Many Blacks live in areas with lower property tax bases, leading to fewer resources to fund public schools. Poor-quality education could lead to low-paying jobs that do not provide health insurance, limiting access to high-quality health care. Low income contributes to more limited housing options, and poor and racial/ethnic minority groups are more likely to live near and be exposed to environmental toxins and pollution^2^. Moreover, racially segregated areas such as American Indian reservations frequently have food deserts and limited transportation options^11^.

Structural racism affects justice and legal systems, diminishing access to opportunity structures with downstream economic, labor, and health effects^2^. Mass incarceration of Black, Latino, and AIAN men results in part from racial profiling and disproportionately higher rates of police stops, arrests, and convictions^2^. Incarceration has profound generational effects on families. Incarceration increases mental and physical health issues from stress and trauma, and it limits resources and causes financial hardship through loss of income and social support. Communities lose economic and social resources of incarcerated members^2^. Families also face social stigma and discrimination, which can isolate them from community and social support. Formerly incarcerated persons have difficulty obtaining jobs. Additionally, children of incarcerated parents are more likely to encounter the criminal justice system themselves, perpetuating a cycle of incarceration^12^.

Structural racism also impacts media, including news and entertainment industries, with synergistic effects in systems such as immigration and foreign policy^2^. The media can perpetuate racism and discrimination by reinforcing stereotypes and biased portrayals of minority groups, which can shape public perception negatively. Historically, anti-Asian immigration laws dating back to the Chinese Exclusion Act of 1882 have been accompanied by media tropes otherizing Asians as “perpetual foreigners” and the “yellow peril.”^2^Modern-day terms such as “kung flu” build on prior racist imagery of Asians as vectors of disease. American foreign policy such as the Vietnam War and Iraq War has harmed many immigrants. Support for refugees and pathways to citizenship has been limited, and discriminatory surveillance and profiling policies intended for national security have targeted MENA^2^. Accompanying media, language and representation frequently dehumanize and create negative stereotypes. For example, Hollywood often portrays diverse MENA persons as Muslim terrorists who oppress women^2^. Words and stories matter. Negative messages and imagery directly stigmatize and traumatize individuals and communities and create subconscious narratives that influence beliefs and attitudes about different populations, and their support for anti-racist solutions that increase access to opportunity structures and improve health.

Structural racism is also perpetuated by aggregating data classification of racial and ethnic populations, which masks inequities among subpopulations. For example, “Hispanic” classification was only introduced as part of the US census in 1980, after a 1976 mandate by Congress. It was included as an ethnic categorization and defined as “Americans [who] identify themselves as being of Spanish-speaking background and trace their origin or descent from Mexico, Puerto Rico, Cuba, Central and South America, and other Spanish-speaking countries.” This broad classification makes invisible the dire health, education, and economic conditions of Hispanic subgroups—specifically Mexican American and Puerto Ricans—who share involuntary historical incorporation in the USA^2^. These two groups have disproportionately experienced long-standing intergenerational life course disadvantages due to US policies, such as segregation and English-only laws and practices, which have created a significant wealth gap. Lack of disaggregating racial and ethnic categories also makes invisible inequities among Asian subgroups, such as for some disadvantaged Southeast Asian populations^2^. Failure to disaggregate Native Hawaiians or Pacific Islanders from more numerous Asians also masks their inequities^2^.

AIAN peoples have a history of unique experiences resulting in health challenges and inequities^2^. Nearly 700 Tribes remain in the USA, with 574 federally recognized. With unique political status, they celebrate their own histories, cultures, languages, and practices enduring centuries of efforts toward genocide including systematic limits on basic human rights, subsequent relocation, exposure to disease, enslavement, violence, boarding schools, forced sterilization, and persistent socio-economic disadvantages^2^. Data genocide removes AIAN peoples from data, making their problems invisible. It has taken hundreds of years for the US government to acknowledge and apologize for boarding school atrocities, let alone substantially remedy their effects. AIAN peoples experience the intersectional effects of poverty and racism including lack of access to high-quality health care, exposure to trauma, and disproportionate exposure to extreme weather events and toxins resulting in increased risk for higher rates of chronic conditions^13^. Historically, the Indian Health Service (IHS) has been funded at only half the per capita rate of other key health systems. Loss of access to land, sacred sites, and traditional medicines including ceremonial practices disrupts key methods AIAN peoples use to sustain their well-being and the health of their communities. Health systems do not always prioritize culturally safe care for AIAN peoples. Justified mistrust of colonial health systems persists in Tribal communities due to a history of abuse and research misconduct, though Tribes do prioritize the health of their citizens^14^.

Health care is not immune to structural racism. For example, structural racism influences payment and financing of the US health care system, resulting in fewer resources for health care delivery organizations that serve racial/ethnic minority populations, affordability problems for many patients, and weak accountability of systems for the health and well-being of persons and communities^2,7,15^. State variation in the resourcing of the Medicaid program has roots in the racial politics of the 1960 s when Medicaid was founded, and today’s underfinancing harms poor people, regardless of their skin color^2^.

## CROSS-CUTTING ACTIONABLE SOLUTIONS

The book outlines cross-cutting actionable solutions to address structural racism to advance equity in health, well-being, and ecosystems. Some solutions could be implemented now, and some are more feasible in the medium and long term. These solutions highlight unfair structures rather than perceived deficits in individuals. Contrary to some attitudes, this mind shift recognizes that the major root cause of health inequities for racial/ethnic minority populations is structural racism and the systemic barriers it creates, rather than assumed personal failings of individuals. Tackling structural and systemic racism in social (Table 1) and health care (Table 2.) systems can improve health outcomes for all^2^. Key principles include partnering with communities and recognizing their expertise, mobilizing community assets, and respecting Indigenous sovereignty (Table 3)^2^. Recent apologies from the US government regarding past atrocities show it is possible for a new era to begin toward healing the relationship with AIAN peoples. Despite past criminalization of AIAN cultural practices, policymakers have taken steps to support their intrinsic value. For example, in 2024, the Centers for Medicare and Medicaid Services approved Sect. 1115 waivers for five states, including Alaska, to pilot Traditional Healing programs. These waivers will support AIAN peoples to practice their holistic understanding of health, balancing spiritual, mental, physical, and emotional forces.

**Table 1:** Actionable Solutions to Address Structural Racism to Advance Health Equity in Social Systems*

**Economic Infrastructure Investments** • Invest in economic development, food security, affordable housing, public transportation, and rural broadband • Create built environments that promote physical activity, ease stress, enhance green space, and reduce vulnerability to severe weather and climate change • Invest in renewable energy infrastructure, particularly in the Oceania territories and freely associated states, to benefit all generations • Address economic insults of the past by providing economic and land reparations • Rectify the racial wage gap and wealth divide • Increase access to home ownership • Increase access to banks and lending institutions in marginalized communities • Increase representation of people from a diverse range of races and ethnicities in leadership across sectors, including government, academia, and healthcare
**Safety Net Investments for Marginalized Communities** • Increase enrollment of those eligible for assistance programs and expand access to overlooked marginalized groups • Design assistance programs to encourage financial security, savings, and economic advancement • Support subsidies for childcare and reduce student loan debt
**Labor/Employment Investments** • Develop and enforce fair working conditions for low-wage workers • Expand paid family leave • Increase federal minimum wage, with built-in cost-of-living increases • Make the Earned Income Tax Credit permanent and expand eligibility • Protect the right to unionize • Increase training for 21st-century jobs • Expand educational opportunities for trade jobs • Improve access to job benefits like health insurance in the gig economy
**Education** • Restructure how education is financed in the U.S. to eliminate inequities • Improve the quality of schooling from early childhood through post-secondary education for all • Reduce student loan debt • Increase investments in education scholarships for students across underrepresented populations • Incorporate accurate history of racial/ethnic minority populations into general educational curricula • Invest in community colleges and vocational schools as pathways to good-paying employment, including the trades • Invest in educational pipelines into the health and science, technology, engineering, and mathematics (STEM) professions for students from underrepresented populations
**Justice and Civil Rights Law** • Ban racial profiling and prevent over-policing • Develop routine public reporting databases • Implement sensible firearm policy • Increase civil rights funding • Create systems and databases to monitor violations of civil rights • Rebalance public investments from incarceration and detention to strengthening communities and families • Restore disparate impact discrimination claims, without having to prove discriminatory intent • Remove barriers to voting
**Media** • Promote accurate representative narratives of heterogeneous communities rather than narratives that use harmful stereotypes • Increase representation in the media/entertainment workforce, including content creators • Counter the representations that members of particular populations are harmful and problematic • Counter the myth that whites are superior to others • Highlight that poor and disadvantaged whites benefit from equitable social and healthcare policies
**Immigration and Foreign Policy** • Provide pathways to citizenship • Relieve immigrant visa backlogs • Remove barriers to citizenship • Eliminate English-only laws and promote language access in voting and all public services • Address policies such as the Patriot Act that create unfair “national security risk” biases against populations such as Middle Eastern or North African (MENA) and Chinese Americans
**Data, Science, and Information** • Disaggregate race/ethnicity data into more granular categories (e.g., specific Asian and Hispanic ethnicities) • Create meaningful categories that accurately capture heterogeneous lived experiences (e.g., Separate Asians and Native Hawaiians or Pacific Islanders, and collect ethnicity data for them) • Embrace the diversity of experiences and potential solutions specific to each community's opportunities and constraints using new and innovative research methods, building on participatory action research

*See *Systems That Impact Population Health: Past and Present* for more details^2^

Table 1 is modified by permission from a version of the content in Clarke I, Warne D, Sharif MZ, et al, eds. *Systems That Impact Population Health: Past and Present*. American Public Health Association Press; 2025

**Table 2.** Actionable Solutions to Address Structural Racism to Advance Health Equity in Health Care Systems*

**Ensure Access to Health Care** • Redesign healthcare financing to expand access to care for marginalized populations • Expand Medicaid and individual non-group market health insurance • Expand support for health insurance navigators and assisters • Increase requirements for employers to offer health insurance, including increasing the enrollment of small businesses in public insurance options • Eliminate barriers to insurance based on immigration status • Repeal any public charge laws that consider a noncitizen’s probability of depending on the government for subsistence when determining whether to grant a green card or visa • Increase Medicaid reimbursement rates so that healthcare delivery organizations have support and incentives to care for Medicaid beneficiaries rather than limit their care
**Implement policies supporting and incentivizing equitable healthcare tailored to meet the needs of all populations** • Support healthcare services that are patient-, family-, and community-centered, including proactive engagement of patients, families, and caregivers during care • Provide trauma-informed care • Clarify requirements and increase enforcement of language access in all healthcare programs and services, including ensuring access to qualified healthcare interpreters and translations of written materials • Expand the role of community health workers (CHWs) and patient advisers to mitigate access, cultural, and structural barriers to healthcare systems • Increase health workforce diversity and ensure federally-funded training and diversity programs create sustainable employment opportunities for underrepresented groups • Increase cultural humility and health equity training for healthcare professionals and staff • Strengthen mental health and substance use disorder services to meet patients where they are • Address health-related social needs such as food insecurity, housing insecurity, and transportation, and structural social drivers of health • Facilitate coalition building and organizing by partnering with communities • Reform payment systems to support and incentivize wellness overall, disease prevention, and care specifically tailored to meet the medical and social needs of all populations • Provide more funding and support to under-resourced healthcare delivery organizations that frequently provide uncompensated or under-compensated care serving racial/ethnic marginalized communities • Increase racial/ethnic diversity in the healthcare workforce, as well as patient-provider racial/ethnic concordance, co-located community healthcare services/whole-person care, team-based care, and care delivery in the language of preference • Develop sustainable models for value-based payment and alternative payment models that reward high-quality, equitable care and outcomes and social return on investment • Focus on structural changes to systems rather than perceived deficits of individuals (e.g., increase after-hours appointment availability for “nonadherent” patients who miss appointments because they cannot get time off from work)

*See *Systems That Impact Population Health: Past and Present* for more details^2^

Table 2. is modified by permission from a version in Clarke I, Warne D, Sharif MZ, et al, eds. *Systems That Impact Population Health: Past and Present*. American Public Health Association Press; 2025

**Table 3:** Actionable Solutions to Address Structural Racism to Advance Health Equity Through Indigenous Sovereignty*

**Land and Ecosystem** • Remove/revise policies that prohibit customary usage and access to ancestral lands and waters by lineal descendants of traditional custodians and residents • Include Indigenous peoples in all levels of systems that protect and value intact ecosystems and intergenerational relationships and that address climate change • Increase funding opportunities for sustainable economic development and renewable energy infrastructure in all Oceania territories and freely associated states
**Healthcare** • Fully fund the Indian Health Service (IHS) • Provide initiatives to design and implement culturally safe and respectful care that understands historical perspective, childhood and intergenerational trauma, and circumstances unique to Indigenous peoples • Support traditional healing practices • Improve coordination across IHS/Tribal/Urban Indian health programs and public/private sector health systems • Develop an available, accessible, affordable, and competent workforce that integrates community voices and AIAN traditions into culturally sensitive care • Incorporate midwives, social workers, mental health counselors, doulas, AIAN traditional healers, knowledge bearers, birth workers and peers, community health workers, and physician extenders into care of Indigenous people • Expand digital and telehealth in resource-limited areas to supplement existing care resources but not as a substitute for care and to provide sufficient resources to these areas
**Economic investments** • Invest in economic infrastructure in AIAN and Native Hawaiian or Pacific Islander (NH/PI) communities. Promoting banking, business development, wireless, and economic sovereignty on reservations, Hawai’i, and U.S. Pacific Island territories is necessary to promote wellness in tribal communities • Implement culturally-rooted and sustainable community-based management systems • Approach economic development as a public health intervention • Develop a system to protect and value intact ecosystems
**Education** • Incorporate accurate AIAN and NH/PI history and cultural values into general educational curricula—Culturally competent education should be reflected in both curricula and personnel • Provide funding and resources to facilitate the universal teaching of Indigenous languages and decolonized history in public schools in the state of Hawai’i, the territories of Guam and American Samoa, the Commonwealth of the Northern Marianas Islands, and the signatory nations of the Compact of Free Association • Invest in AIAN and NH/PI schools and tribal colleges • Foster intergenerational relationships with elders and knowledge bearers
**Data** • Ensure tribal sovereignty and ownership and control of data • Disaggregate the NH/PI category from the broader Asian category; disaggregate tribal data

*See *Systems That Impact Population Health: Past and Present* for more details^2^

Table 3 is modified by permission from a version in Clarke I, Warne D, Sharif MZ, et al, eds. *Systems That Impact Population Health: Past and Present*. American Public Health Association Press; 2025

Public and private policymakers need to intentionally address inequities stemming from structural racism. In health care, the Centers for Medicare and Medicaid Services Framework for Health Equity 2022–2032 has provided a guide for implementing promising health equity initiatives. However, the Trump administration is attacking diversity, equity, and inclusion initiatives^9^. Much more needs to be done to expand access to care for marginalized populations, increase Medicaid reimbursement rates^15^, support patient-centered health care tailored to meet the medical and social needs of patients^6^, and develop sustainable models for value-based payment and alternative payment models that reward high-quality, equitable care and outcomes and social return on investment^6,7^. These interventions benefit all people and communities, not just persons from racial/ethnic minority groups. The Indian Health Service should be fully funded^2^.

## ADDRESSING STRUCTURAL RACISM BENEFITS EVERYONE

Successfully addressing structural and systemic racism to improve health equity is not a pipedream. Americans widely support giving everyone a fair and just opportunity for health, and the proposed solutions for social and health care systems in the book are concrete and specific, and would markedly improve equity in health, well-being, and cultural ecosystems^2^. Economic costs to the USA of racial and ethnic health inequities were estimated to be $421–451 billion in 2018^16^, and the human cost in deaths, longevity, and quality of life is immense. Health inequities need not be a scourge if the nation takes action to address structural and systemic racism. Advancing health equity is not a zero-sum game^4^. Improving social and health care systems helps the poor, marginalized white populations, people of color, and the nation overall^2^. We all gain by addressing structural racism.

## Declarations

### Disclaimer

The thoughts and ideas expressed in this article are those of the authors and do not necessarily represent the views or policies of their employers or other organizations associated with the authors.
